# MicroRNA profiling to inform disease classification, severity, and treatment response in pediatric pulmonary hypertension

**DOI:** 10.1152/ajpheart.00622.2024

**Published:** 2024-11-26

**Authors:** Michael A. Smith, Sam Chiacchia, Jason Boehme, Sanjeev A. Datar, Emily Morell, Roberta L. Keller, Amy Romer, Elizabeth Colglazier, Claire Parker, Jasmine Becerra, Jeffrey R. Fineman

**Affiliations:** 1 Division of Pediatric Critical Care, Department of Pediatrics, University of California, San Francisco, California, United States;; 2 Division of Pediatric Pulmonary Hypertension, Department of Pediatrics, University of California, San Francisco, California, United States;; 3 Department of Emergency Medicine, Stanford University, Palo Alto, California, United States;; 4 Division of Neonatology, Department of Pediatrics, University of California, San Francisco, California, United States;; 5 Division of Pediatric Critical Care, Department of Pediatrics, Children’s Hospital of Philadelphia, Pennsylvania, United States

**Keywords:** microRNA, pediatrics, pulmonary hypertension

## Abstract

Pediatric pulmonary hypertension is a heterogeneous disease associated with significant morbidity and mortality. MicroRNAs have been implicated as both pathologic drivers of disease and potential therapeutic targets in pediatric pulmonary hypertension. We sought to characterize the circulating microRNA profiles of a diverse array of pediatric patients with pulmonary hypertension using high-throughput sequencing technology. Peripheral blood samples were drawn from patients recruited at the time of a clinically indicated cardiac catheterization, and microRNA sequencing followed by differential expression and target/pathway enrichment analyses were performed. Among 63 pediatric patients with pulmonary hypertension, we identified specific microRNA signatures that uniquely classified patients by disease subtype, correlated with indicators of disease severity including invasive hemodynamic metrics, and changed over the course of treatment for pulmonary hypertension. These microRNA profiles include a number of specific microRNA molecules known to function in signaling pathways critical to pulmonary vascular biology and disease, including transforming growth factor-β (TGF-β), VEGF, PI3K/Akt, cGMP-PKG, and HIF-1 signaling. Circulating levels of miR-122–5p, miR-124–3p, miR-204–5p, and miR-9–5p decreased over the course of treatment in a subset of patients who had multiple samples drawn during the study period. Our findings support the further investigation of specific microRNAs as mechanistic mediators, biomarkers, and therapeutic targets in pulmonary hypertension.

**NEW & NOTEWORTHY** We present novel insight into the circulating microRNA profiles of pediatric patients with pulmonary hypertension. Our findings support the utility of microRNAs as both useful biomarkers of disease severity and potential therapeutic targets in pediatric pulmonary hypertension.

## INTRODUCTION

Pulmonary hypertension (PH) is a debilitating disease of the cardiopulmonary system that affects both adults and children. PH is defined as an increase in pulmonary artery pressure (PAP) ≥ 20 mmHg with a pulmonary vascular resistance (PVR) ≥ 3 Wood units and a pulmonary capillary wedge pressure ≤ 15 mmHg to specify precapillary PH (1). A wide range of pediatric diseases can lead to endothelial dysfunction, abnormal smooth muscle proliferation, and pulmonary vascular remodeling that contribute to the abnormal hemodynamics of PH (2). The World Symposium on Pulmonary Hypertension (WSPH) Pediatric Task Force in 2018 highlighted the significant differences in disease epidemiology, pathophysiology, and response to treatment in pediatric compared with adult PH, stressing the need for improved disease phenotyping and encouraging efforts to more deeply explore the unique underlying pathobiology of pediatric PH (1, 3).

The function of microRNAs (miRNAs) in PH has been increasingly investigated in recent years. miRNAs are small noncoding RNA molecules that can negatively regulate numerous target genes via either transcript degradation or repression of mRNA translation (4). In PH, miRNAs have been studied both for their role in disease pathogenesis and as potential therapeutic targets (5, 6). Several studies in pediatric patients have identified specific miRNAs through microarray profiling whose circulating levels correlate with invasive hemodynamic metrics (7) or whose differential expression is associated with PH development and longitudinal outcomes in specific subsets of patients with PH (8, 9).

A comprehensive profiling of circulating miRNAs in a diverse array of pediatric patients with PH employing next-generation sequencing techniques is yet to be undertaken. As such, we sought to collect invasive hemodynamic measurements and perform miRNA sequencing on peripheral blood samples from patients managed at our pediatric PH specialty care center. Our investigation had three objectives. First, to assess whether certain circulating miRNAs are differentially expressed in specific subtypes of pediatric PH, potentially offering insight to inform disease pathophysiology and classification. Second, to correlate circulating miRNA levels with clinical outcomes and markers of disease severity and determine whether miRNAs could function as prognostic biomarkers. And finally, to explore how miRNA profiles change with treatment and assess whether miRNA levels could be used as a less invasive means of measuring response to therapy.

## MATERIALS AND METHODS

### Patient Enrollment and Cardiac Catheterization

The study protocol was reviewed and approved by the Institutional Review Board at the University of California, San Francisco. Parents/guardians of patients <21 yr of age were approached for enrollment between June 2016 and November 2021 during preparation for a clinically indicated cardiac catheterization. Patients were eligible for inclusion if they had evidence of PH on prior cardiac catheterization or concern for PH on echocardiography based on right ventricular pressure estimates. All patients enrolled at the time of their first-ever cardiac catheterization had confirmed PH on the study catheterization [mean PAP ≥ 20 mmHg and indexed PVR (PVRi) ≥ 3 Wood units·m^2^]. Written informed consent was obtained. After enrollment, patient demographic and clinical characteristics were collected from chart review, including PH classification and World Health Organization (WHO) functional class at the time of enrollment. Patients were followed for important clinical outcomes of Potts shunt placement, lung transplant, and death. Invasive hemodynamic data were collected from catheterization reports; only clinically indicated measurements were collected for each patient.

### miRNA Sequencing, Library Preparation, and Quantification of Gene Expression

Venous blood was collected at the start of catheterization once vascular access was obtained. Plasma samples were stored at −80°C until RNA isolation. RNA was isolated using the miRNeasy Advanced Kit (QIAGEN) according to the manufacturer’s instructions. Library preparation was done using the QIAseq miRNA Library Kit. After reverse transcription and adapter ligation, the cDNA was amplified using PCR (22 cycles). After PCR, the samples were purified, and quality control was performed using capillary electrophoresis (Tape D1000). The library pools were then sequenced on a NextSeq (Illumina Inc.) sequencing instrument according to the manufacturer’s instructions (1 × 75, 2 × 10). Raw data were demultiplexed and FASTQ files for each sample were generated using the bcl2fastq2 software (Illumina Inc.). Samples were sequenced in two batches, which was accounted for in downstream bioinformatic analyses.

The “QIAseq miRNA Quantification” workflow of the CLC Genomics Server with standard parameters was used to map the reads to miRBase version 22. In short, the reads were processed by trimming of the common sequence, unique molecular identifier (UMI), and adapters, followed by filtering of reads with length of <15 nucleotides or length of >55 nucleotides. They were then deduplicated using their UMI. Reads were grouped into UMI groups when they *1*) started at the same position based on the end of the read to which the UMI was ligated, *2*) were from the same strand, and *3*) had identical UMIs. Reads were then mapped to miRBase version 22 and quantified.

### Bioinformatic Analysis

Differential miRNA expression analyses were performed using the EdgeR pipeline in R, version 4.4.1 (10, 11). Data preprocessing included the removal of underexpressed miRNA and trimmed mean of M (TMM) normalization. Preprocessed, normalized expression values were fit to gene-specific binomial linear models, controlling for sequencing batch number, to evaluate differential expression between test groups. Only initial sample data were included, except for analyses assessing miRNA response to treatment. For comparisons between PH subtypes, patients were stratified into groups based on similar disease pathophysiologies underlying their primary PH etiology. Statistical significance for differential expression was defined as a false discovery rate (FDR) of <0.05, generated by Benjamini–Hochberg *P* value correction for multiple hypothesis testing. Differentially expressed miRNA were imported into miRPath v4.0 (12) to identify enriched pathways from the Kyoto Encyclopedia of Genes and Genomes (KEGG) Pathway database (13), specifying target gene union merging using TarBase v8.0 to identify direct targets of the miRNA. If only one miRNA was differentially expressed in an analysis, miRPathDB (14) was queried to identify enriched KEGG pathways. TarBase v9.0 and PubMed were additionally queried to identify additional experimentally validated miRNA-mRNA interactions (15).

### Statistical Analysis

Data are presented as means ± standard deviation, unless otherwise indicated. For analyses correlating miRNA expression levels with continuous catheterization variables, initially, all miRNAs were screened with differential expression analyses regressing miRNA expression against PVRi and mean PAP as continuous variables. For those miRNAs significant in regression modeling for an association with PVRi and/or mean PAP, Spearman correlations were calculated for both PVRi and mean PAP with batch-corrected, log_2_-normalized expression levels. For comparisons of hemodynamic metrics at first and follow-up catheterizations, paired Wilcoxon rank sum tests were used. A *P* value ≤ 0.05 was considered statistically significant.

## RESULTS

### Patient Characteristics

A total of 63 patients were enrolled in the study. Table 1 describes patient demographic and clinical characteristics. The mean age at enrollment was 6.7 ± 5.9 yr. Most patients were primarily classified as Group 1 PH (*n* = 40, 63.4%) as per the WSPH classification system (1), followed by Group 3 (*n* = 21, 33.3%), Group 2 (*n* = 1, 1.6%), and Group 4 (*n* = 1, 1.6%). WSPH classes are broad and often group together diseases with disparate pathophysiologies. For further analyses, patients were stratified into PH subtypes based on similar disease pathophysiology. These included bronchopulmonary dysplasia-PH (BPD, *n* = 12, 19.0%), congenital diaphragmatic hernia/pulmonary hypoplasia-PH (CDH, *n* = 9, 14.3%), congenital heart disease-PH (CHD, *n* = 24, 38.1%), idiopathic/heritable PH (*n* = 15, 23.8%), and other (*n* = 3, 4.8%). Genetic mutations identified in the patients with heritable PH included BMPR2 (*n* = 3), ATP13A3 (*n* = 1), TBX4 (*n* = 1), and GDF2 (*n* = 2) mutations.

Table 2 describes the patient functional status and invasive hemodynamic data at the time of enrollment as well as longitudinal clinical outcomes. At the time of enrollment, 44.4% of the patients were classified as WHO functional class I-II and 33.3% were classified as class III-IV. Over a mean follow-up time of 6.9 ± 1.5 yr, 15 patients (23.8%) received a Potts shunt, underwent lung transplantation, and/or died.

### Differential Expression of miRNAs between Disease Subtypes

**Figure 1.**
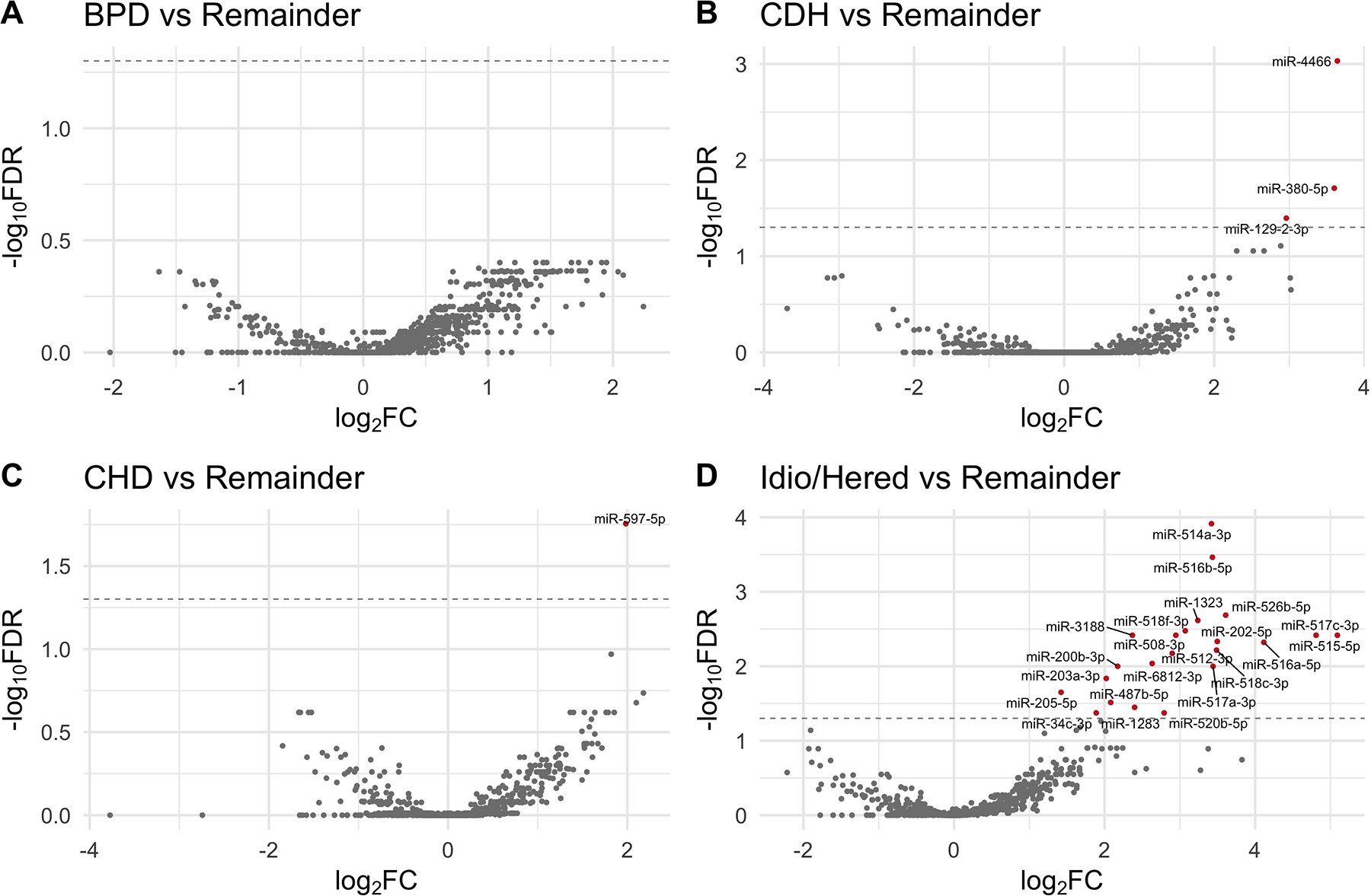
Volcano plots depict the results of differential expression analyses identifying miRNA profiles unique to specific subtypes of pulmonary hypertension. Analyses compared miRNA expression within a specific PH subtype (BPD, *A*; CDH, *B*; CHD, *C*; and idiopathic/heritable, *D*) with the average expression among the remainder of patients with PH. Statistical significance was defined by an FDR-adjusted *P* value < 0.05. BPD, bronchopulmonary dysplasia; CDH, congenital diaphragmatic hernia; CHD, congenital heart disease; FC, fold change; FDR, false discovery rate; Idio/Herit, idiopathic/heritable; PH, pulmonary hypertension.

In differential expression analyses, several miRNAs were found to be significantly differentially expressed in patients with specific subtypes of PH when compared with the average expression levels among the remaining patients with PH (Fig. 1, Supplemental Table S1). Patients with CDH-PH demonstrated increased circulating levels of miR-4466, miR-380–5p, and miR-129-2-3p, and patients with CHD-PH had increased levels of miR-597–5p (FDR < 0.05). Twenty-two miRNAs were significantly upregulated in patients with idiopathic/heritable-PH (FDR < 0.05).

Table 3 highlights key validated target mRNA genes and biological pathways modulated by the differentially expressed miRNAs (see Supplemental Fig. S1 for detailed pathway enrichment results). The significantly upregulated miRNAs in patients with CDH and CHD have been shown to function in the interaction between cells and the extracellular matrix, fluid shear stress, and cancer biology. The miRNAs that differentiated patients with idiopathic/heritable PH play roles in several signaling pathways that are well described to be dysregulated by the commonly described mutations associated with PH, including transforming growth factor-β (TGF-β) signaling, VEGF signaling, and PI3K/Akt signaling, among others.

### Correlating miRNA Levels with Disease Severity

We next assessed whether miRNA levels were associated with longitudinal outcomes or correlated with markers of disease severity within the different subtypes of patients with PH. There were no miRNAs differentially expressed at the time of study enrollment between those who eventually went on to require a Potts shunt, lung transplant, or died and those who did not. However, WHO functional class at the time of enrollment was associated with differential expression of two miRNAs. Compared to patients with WHO functional class I-II, patients with worse functional class (class III-IV) demonstrated decreased expression of MiR-3117–3p and increased expression of MiR-380–3p (log_2_FC = −2.8 and FDR = 0.005, log_2_FC = 2.7 and FDR = 0.009, respectively).

**Figure 2.**
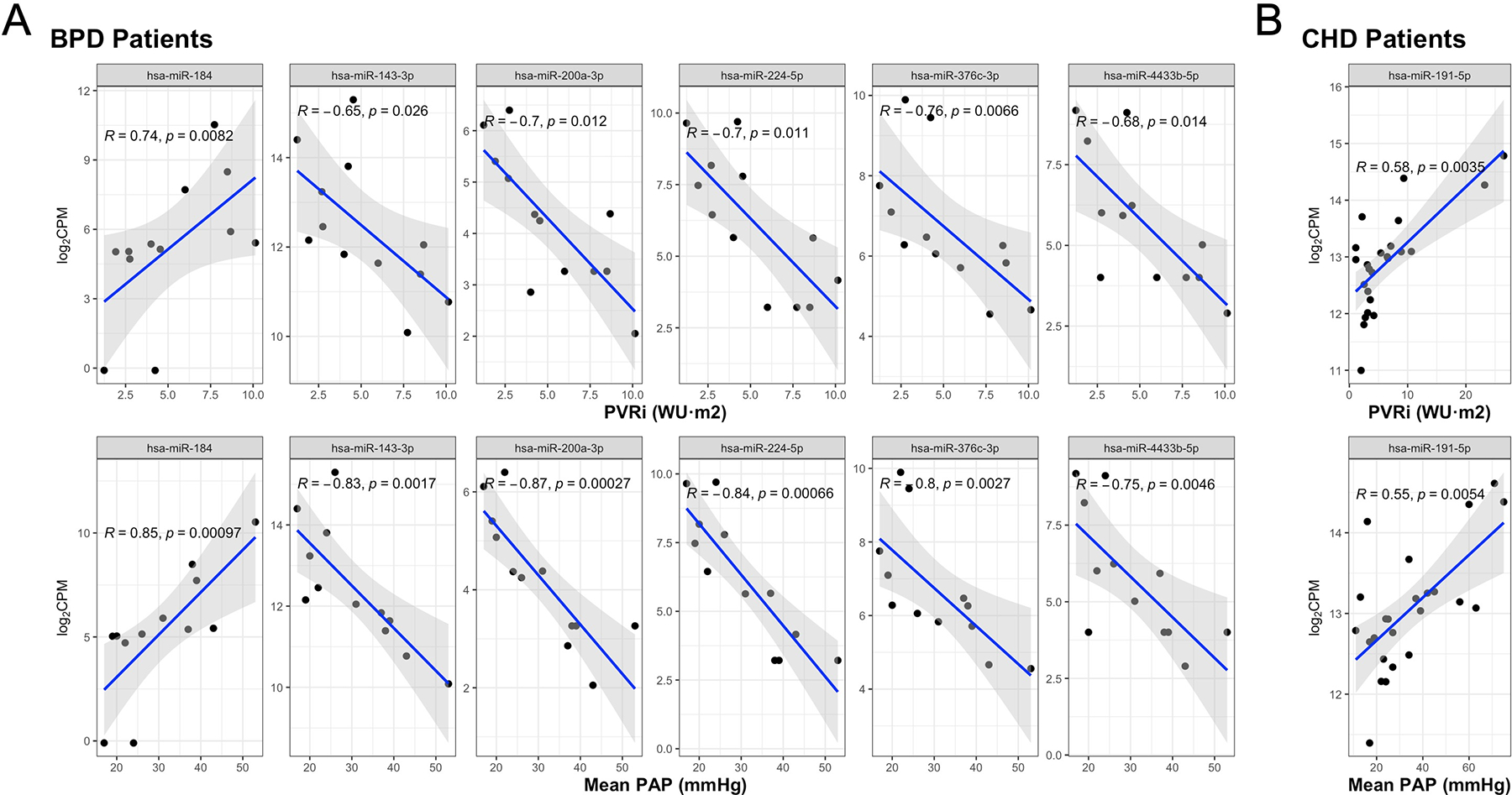
Correlation plots demonstrate significant associations between circulating miRNA levels and PVRi and mean PAP in patients with BPD (*A*) and patients with CHD (*B*). Correlation coefficients (*R*) and *P* values are derived from Spearman correlations. BPD, bronchopulmonary dysplasia; CHD, congenital heart disease; CPM, counts per million; PAP, pulmonary artery pressure; PVRi, indexed pulmonary vascular resistance.

Among patients with BPD, expression levels of six miRNAs were found to correlate significantly with both PVRi and mean PA pressure (Fig. 2*A*). MiR-184 increased with increasing PVRi and mean PA pressure, whereas miR-143–3p, miR-200a-3p, miR-224–5p, miR-376c-3p, and miR-4433b-5p all decreased with increasing PVRi and mean PA pressure. Among patients with CHD, miR-191–5p was significantly positively correlated with PVRi and mean PA pressure (Fig. 2*B*). No miRNAs were identified that significantly correlated with invasive hemodynamics among patients with CDH, idiopathic/heritable, or other PH subtypes.

### miRNA Response to Treatment

Finally, we assessed the change in circulating miRNA levels that occurred over the course of treatment in a subset of patients who underwent two catheterizations with repeat sample collections during the study period. Table 4 describes the nine patients’ disease classifications and subtypes as well as therapies prescribed at the time of each sample collection. The interval between collections ranged from 6 to 60 mo (median = 12 mo, interquartile range = 8–12 mo). All nine patients had either maintenance or escalation of PH therapies over this time interval.

**Figure 3.**
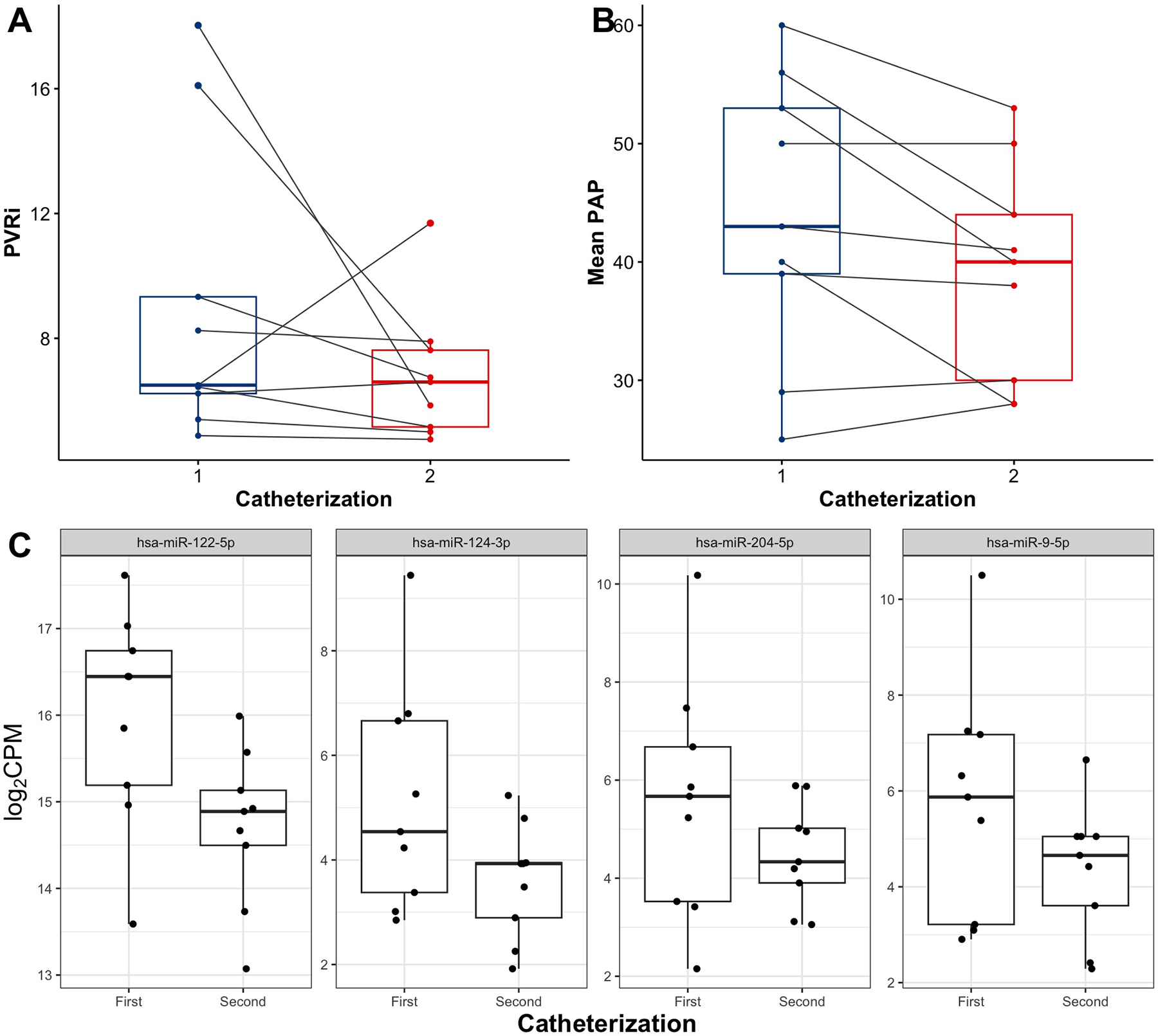
Changes in invasive hemodynamics (PVRi, *A*; mean PAP, *B*) and circulating miRNA expression levels (*C*) between first and second catheterizations among those patients with multiple catheterizations and blood sample collections during the study period. Levels of miRNAs miR-122–5p, miR-124–3p, miR-204–5p, and miR-9–5p decreased significantly between first and second catheterizations (FDR < 0.05). CPM, counts per million; FDR, false discovery rate; PAP, pulmonary artery pressure; PVRi, indexed pulmonary vascular resistance.

Figure 3, *A* and *B*, depicts the patients’ hemodynamic data at initial and repeat catheterizations. Although the group’s median PVRi (6.5 Wood units·m^2^ vs. 6.6 Wood units·m^2^) and mean PA pressures (43 mmHg vs. 40 mmHg) were similar between the two catheterizations, most patients did show some improvement in their hemodynamics over the interval period (*P* = 0.16 and *P* = 0.09 for paired analyses of PVRi and PA pressure, respectively).

**Figure 4.**
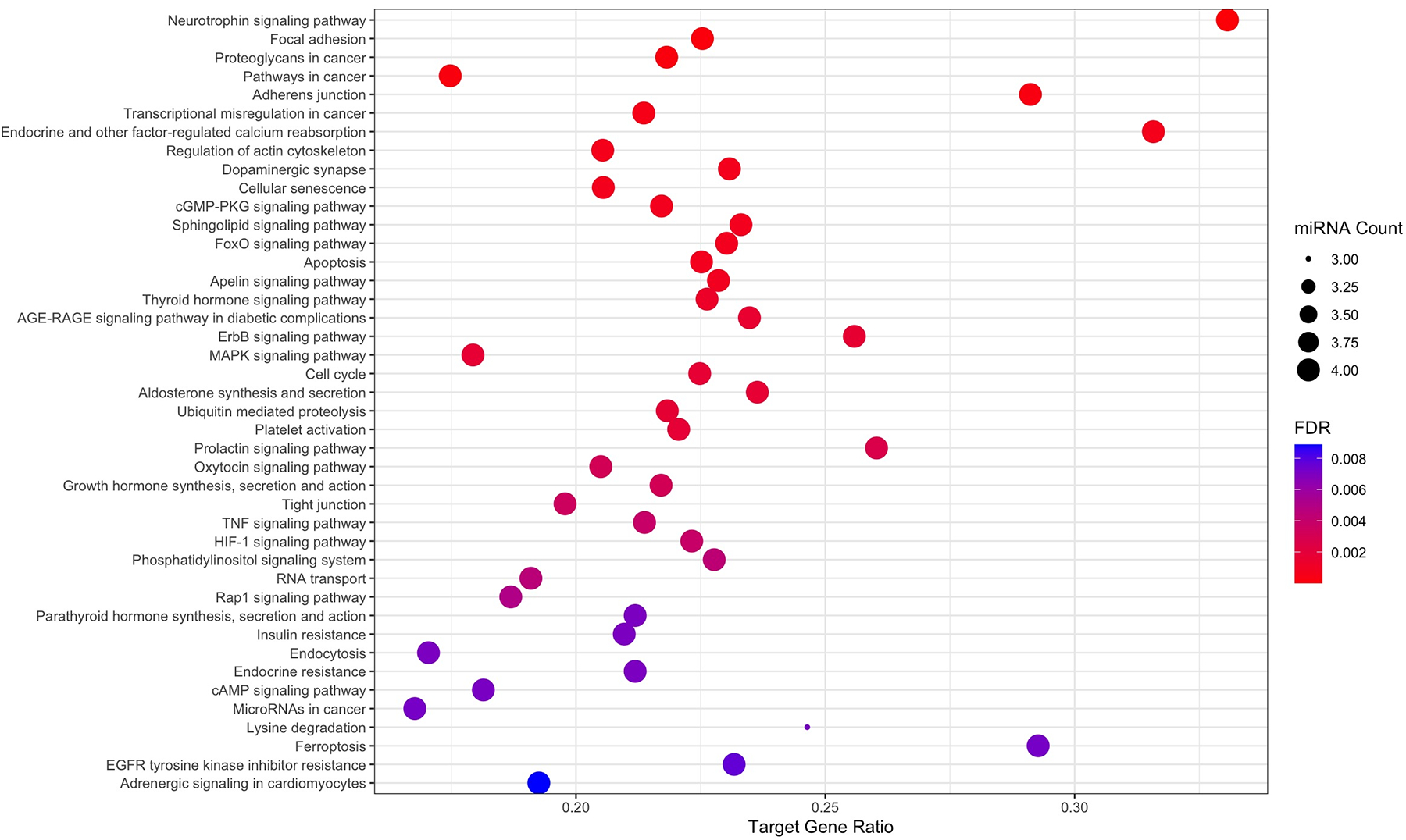
Results of miRPath v4.0 KEGG pathway enrichment analyses assessing the four miRNAs identified to decrease significantly over the course of treatment in our cohort (miR-122–5p, miR-124–3p, miR-204–5p, and miR-9–5p). Enrichment analyses assessed whether the experimentally validated target mRNAs of the input miRNAs enrich specific biological pathways, thus potentially implicating these pathways as modulated over the course of PH treatment. miRNA count indicates the number of input miRNAs that are implicated in each pathway. Target gene ratio denotes the ratio of validated gene targets of input miRNAs to the total number of genes annotated to the pathway. False discovery rates (FDR) are derived from *P* values determined from Fisher’s exact tests assessing term overrepresentation in targets of the input miRNAs.

Four miRNAs were identified to have significantly lower expression in the second catheterization compared with the first, namely, miR-122–5p, miR-124–3p, miR-204–5p, and miR-9–5p (FDR < 0.05, Fig. 3*C*). Supplemental Table S2 lists the experimentally validated mRNAs documented in Tarbase v9.0 that are directly targeted by these miRNAs. These miRNAs and their mRNA targets significantly enrich a number of biological pathways, a selection of which are shown in Fig. 4. Several of these pathways, including cGMP-PKG signaling (65, 66), HIF-1 signaling (67–69), and MAPK signaling pathways, are known to play key roles in PH pathophysiology and are targeted by PH-directed therapeutics.

## DISCUSSION

In this study, we present a detailed description of the circulating miRNA profiles of pediatric patients with PH. We have successfully identified specific miRNA signatures that are unique to certain PH subtypes, that correlate with disease severity, and that change over the course of the treatment of PH. These findings help to develop our understanding of the role of miRNAs in pulmonary vascular dysfunction and present opportunities to further explore miRNAs as biomarkers of disease severity and therapeutic targets in pediatric PH.

PH is associated with many diverse pediatric diseases, each of which may have a unique pathobiology contributing to the development of pulmonary vascular disease. Our findings associating certain circulating miRNAs with specific disease subtypes can help to unveil some of the differing pathologic mechanisms of PH. For example, miR-516b-5p, one of the most significantly upregulated miRNAs in patients with idiopathic/heritable PH in our cohort, has been previously shown to directly modulate TGFβR2 expression in endothelial cells (39). Dysregulation of the balance between TGF-β and bone morphogenetic protein (BMP) signaling is a well-recognized driver of PH, especially among patients with idiopathic/heritable PH in whom BMPR2 mutations are the most commonly recognized genetic etiology of PH (70). Our data suggest a potential role for miR-516b-5p in the dysregulated TGF-β signaling in these patients, either as directly pathologic or in response to the imbalance. On the contrary, among patients with CDH in our cohort, miR-4466 was found to be significantly overexpressed. miR-4466 downregulates the SKI proto-oncogene, which functions as a repressor of TGF-β signaling (71). Experimental models of neonatal lung disease have previously shown a role for increased TGF-β signaling in dysplastic lung development (72). Although TGF-β signaling appears to be implicated in both subtypes of PH in our cohort, the mechanistic underpinnings appear distinct, at least in relation to miRNA biology. Not only do these insights present new opportunities to better characterize disease mechanisms, but they may also be used for more detailed phenotyping of disease subgroups and to better inform classification and improve precision medicine efforts.

In identifying specific miRNAs that correlate with invasive hemodynamic measures of disease severity, we add to a small body of work that is already validating circulating miRNA levels as biomarkers of PH disease activity (7, 9, 73–75). The current gold standard for PH disease assessment is invasive hemodynamic evaluation via cardiac catheterization. Less invasive metrics such as echocardiographic measures and serum natriuretic peptide levels are limited by accuracy and reproducibility issues and often do not reflect subtle differences in pulmonary vascular health (76). Blood sampling for miRNA expression offers an additional less invasive and potentially more informative metric. In both patients with BPD and those with CHD, we identified miRNAs with circulating levels that correlated significantly with PVRi and mean PA pressure. Prior work helps to validate the relationships between these specific miRNAs and PH hemodynamics (77, 78). Among patients with BPD in our cohort, miR-143–3p was negatively correlated with PVRi and mean PA pressure. This miRNA has previously been shown to robustly reduce smooth muscle cell proliferation in the lungs, a key pathologic feature of PH (77). In our patients with CHD, miR-191–5p positively correlated with PVRi and mean PA pressure. This same miRNA was found to be significantly upregulated in the hypertrophied right ventricle of an ovine model of CHD-associated pulmonary hypertension (78). Interestingly, circulating miRNA expression levels did not correlate with hemodynamic metrics in CDH, idiopathic/heritable, or other PH subtypes. This may be related to greater heterogeneity of disease among these subtypes in our cohort. Alternatively, our findings may relate to the underlying differences in disease pathophysiology between these groups, which deserves further exploration.

We identified four miRNAs whose circulating levels significantly decreased over the course of treatment for PH, namely, miR-122–5p, miR-124–3p, miR-204–5p, and miR-9–5p. These miRNAs enrich many biological pathways that are closely tied to PH pathophysiology. The potential implications of these findings are manyfold, including the possibility of using these miRNA levels as markers of response to treatment, to better understand therapeutic mechanisms, and to inform future efforts to develop targeted therapeutics. Three of these miRNAs have already been well described in PH literature (5). miR-124–3p has been shown to affect pulmonary artery smooth muscle, endothelial cell, and fibroblast metabolism and proliferation, with decreased levels seen in the lungs of patients with PH (79–83). Similarly, decreased levels of miR-204–5p have been associated with the proliferative and antiapoptotic phenotype of pulmonary artery smooth muscle cells (84–86) and endothelial-mesenchymal transition in pulmonary artery remodeling (87). Our data showed decreasing levels of circulating miR-124–3p and miR-204–5p over the course of treatment, perhaps reflecting the persistence of this component of the disease process while therapeutics targeted downstream aberrations. On the contrary, miR-122–5p has been previously found to be upregulated in pulmonary microvascular endothelial cells, pericytes, and lung tissue and downregulated in pulmonary artery smooth muscle cells from patients with idiopathic PH and rat models of PH, suggesting a direct role for miR-122–5p signaling in pathologic endothelial and smooth muscle cell proliferation (88). Additional studies have further validated the upregulation of miR-122–5p in lung tissue and circulating blood of patients with PH (89, 90). Decreasing levels in our patients over the course of treatment of miR-122–5p as well as miR-9–5p, which has been implicated in airway smooth muscle cell proliferation and apoptosis in asthma (91), may reflect a true treatment effect in our cohort. A better understanding of the roles these specific miRNAs play in driving PH pathophysiology or ameliorating PH symptoms may be gained from future targeted assessments of their correlations with treatment and clinical changes in patients with PH.

Our study has several caveats and limitations worth noting. It is important to note that our miRNA levels were measured in peripheral blood, and thus, the miRNA source may not be from the heart or lungs but released from other systems as a response to PH. There are many factors that contribute to disease heterogeneity among our cohort that could not be accounted for in our analyses, including age, developmental stage of the lungs/pulmonary vessels, and additional therapeutic interventions such as surgical correction of congenital heart lesions. Many of our patients also had numerous factors that could have contributed to the development of PH. We determined primary PH etiology from clinical consensus among our group, although it is not possible to perfectly stratify and account for all factors that contributed to disease in each of our patients. Combined with our relatively small sample size, this clinical heterogeneity may have presented bias that could obscure true signals in the miRNA data and reinforces the need for additional studies to corroborate our findings. We were additionally limited by the lack of data from a cohort of patients without PH, thus limiting our interpretations of circulating miRNAs as biomarkers to patients with established PH. Future investigations should strive to incorporate normal control samples and collect detailed clinical data to combine more comprehensive phenotyping with the rich genomic data obtained from next-generation sequencing.

In conclusion, we present a detailed profiling of the circulating miRNA profiles of a diverse set of pediatric patients with PH. The specific miRNAs identified to differentiate PH disease subtype, correlate with disease severity, and change with treatment have validated roles in a number of biological pathways critical to pulmonary vascular disease. There is ample evidence to support the further investigation of these miRNAs as mechanistic mediators, biomarkers, and therapeutic targets in PH.

## Supplementary Material

Target mRNA of miRNA improved after treatment

Differential miRNA expression results

Supplemental Figure 1

## Figures and Tables

**Table 1.** Patient Characteristics

		Total, 63
**Age at first catheterization, years (mean (SD))**		6.70 (5.93)
**Sex, male**		21 (33.3)
**Race/Ethnicity**	White/Caucasian	26 (41.3)
	Asian	11 (17.5)
	Black or African American	6 (9.5)
	Hispanic or Latino	13 (20.6)
	Native Hawaiian or Other Pacific Islander	3 (4.8)
	Other	4 (6.3)
**Treatment naïve**		10 (15.9)
**Primary Classification**	**Group 1**	40 (63.4)
	*Idiopathic PAH*	*8 (12.7)*
	*Heritable PAH*	*7 (11.1)*
	*Congenital Heart Disease PAH*	*24 (38.1)*
	*Chronic liver disease*	*1 (1.6)*
	**Group 2**: Left atrial or ventricular disease	1 (1.6)
	**Group 3**	21 (33.3)
	*Bronchopulmonary dysplasia*	*12 (19.0)*
	*Congenital diaphragmatic hernia*	*8 (12.7)*
	*Congenital pulmonary hypoplasia*	*1 (1.6)*
	**Group 4**: Chronic thromboembolic	1 (1.6)
**Pulmonary vein stenosis**		9 (14.3)
**Trisomy 21**		7 (11.1)

Patient characteristics were obtained at the time of enrollment in the study. Treatment naïve indicates no phosphodiesterase 5 inhibitors, endothelin receptor antagonists, or prostaglandins prior to enrollment. Values indicate n (%) unless otherwise indicated. PAH, pulmonary arterial hypertension. SD, standard deviation.

**Table 2.** Functional Class, Hemodynamic, and Outcome Data

		Total, 63
**WHO Functional Class**	I	4 (6.3)
	II	24 (38.1)
	III	20 (31.7)
	IV	1 (1.6)
	Missing	14 (22.2)
**Hemodynamics**	Mean RA Pressure	7.00 [5.00, 8.00]
	Systolic PA Pressure	48.00 [32.00, 65.00]
	Diastolic PA Pressure	19.00 [14.00, 28.50]
	Mean PA Pressure	31.00 [22.50, 43.00]
	Mean Systemic Arterial Pressure	64.00 [57.25, 73.75]
	Mean PCWP	10.00 [8.00, 13.00]
	Cardiac Index	3.33 [2.67, 3.99]
	Qp:Qs	1.04 [1.00, 1.36]
	PVRi	5.40 [3.16, 8.34]
**Outcome**	Potts Shunt	3 (4.8)
	Transplant	3 (4.8)
	Death	12 (19.0)
	Potts, Transplant, or Death	15 (23.8)

Functional class and hemodynamic data at the time of study enrollment and longitudinal clinical outcomes are presented. Values indicate n (%) for categorical variables and median [interquartile range] for continuous variables. Pressures are measured in mmHg, PVRi is measured in Wood units indexed to body surface area. Data was missing from 2 patients for mean RAP, 1 patient for mean systemic arterial pressure, 3 patients for mean PCWP, and 30 patients for Qp:Qs. PA, pulmonary arterial. PCWP, pulmonary capillary wedge pressure. PVRi, indexed pulmonary vascular resistance. Qp, pulmonary flow. Qs, systemic flow. RA, right atrial. WHO, World Health Organization.

**Table 3.** Overview of select differentially expressed miRNAs, target genes, and enriched KEGG pathways

PH Subtype	miRNA	Validated Target Genes	Enriched Pathways (FDR < 0.05)	References
CDH-PH	miR-4466	MIF, SKI	Focal adhesion, Adherens junction, Fluid shear stress and atherosclerosis	(44, 45)
	miR-380–5p	BACH1, TEP1, P53, CD276		(46–49)
	miR-129-2-3p	DNMT3B, CCP110, SEMA4C, BZW1, SYK, GABRA1, WIP1, CASP6, CCR2, SOX4, MAP3K7		(50–57)
CHD-PH	miR-597–5p	CXCL5, TEAD1, FOSL2, ELK1	Adherens junction, Proteoglycans in cancer	(58–61)
Idiopathic/Hereditary ^a^	miR-514a-3p	PTPN11, EGFR, PEG3, CTNNB1, CDK2, MC1R, NF1, FOXO4	Ubiquitin mediated proteolysis, Hippo signaling, Pathways in cancer, Cell cycle, p53 signaling, TGF-beta signaling, Adherens junction, Proteoglycans in cancer, PI3K-Akt signaling, FoxO signaling, Focal adhesion, ErbB signaling, MAPK signaling, Ras signaling, Rap1 signaling, Fluid shear stress and atherosclerosis, VEGF signaling	(62–66)
	miR-516b-5p	PDE5A, VEGFA, IFI30, SOSC2, TGFBR2, AKAP2, H6PD, STAT3, KPNA4, ITGA11, SIRT3, FOXO1		(21, 67–74)
	miR-526b-5p	CMYC, FOXP1, ROBO1, SERP1, STMN1, HGF, EIF5A2, CTNNB1		(75–81)
	miR-1323	BMP4, SMAD4, CBLB, IL6, TP53INP1, PABPN1, PDCD4, FRS2, TPD52		(82–88)
	miR-518f-3p	PPARA, TRIP4, MDM2		(89–91)

Overview of miRNAs identified to be differentially expressed in specific subtypes of PH, including experimentally validated target genes and enriched KEGG pathways derived from miRPath v4.0 pathway enrichment analyses.

a The 5 miRNAs with the lowest FDR for idiopathic/hereditary patients in differential expression analyses are displayed here. Pathway enrichment analyses incorporated all 22 miRNA that were significantly overexpressed in idiopathic/hereditary patients. CDH, congenital diaphragmatic hernia. CHD, congenital heart disease. FDR, false discovery rate.

**Table 4.** Patients with repeat catheterization and sample collection during study period

Patient	Primary WSPH Classification	Disease Subtype	Sample Interval (mo)	Therapies at Cath #1	Therapies at Cath #2
1	Group 1	CHD-PH	12	Sildenafil, Ambrisentan	Sildenafil, Ambrisentan
2	Group 1	CHD-PH	12	Sildenafil, Ambrisentan	Sildenafil, Ambrisentan, SC Treprostinil
3	Group 3	CDH-PH	8	Sildenafil, Bosentan, SC Treprostinil	Tadalafil, Bosentan, SC Treprostinil
4	Group 1	Idiopathic	6	None	Sildenafil, Bosentan, SC Treprostinil
5	Group 1	Other (Portopulmonary Hypertension)	12	Sildenafil, INH Treprostinil	Sildenafil, INH Treprostinil
6	Group 1	Idiopathic	60	Sildenafil	Sildenafil
7	Group 1	CHD-PH	12	Tadalafil, Ambrisentan, SC Treprostinil	Tadalafil, Ambrisentan, PO Treprostinil
8	Group 1	Hereditary (GDF2)	12	Tadalafil, Ambrisentan, SC Treprostinil	Tadalafil, Ambrisentan, SC Treprostinil
9	Group 1	Hereditary (BMPR2)	8	None	Tadalafil, Ambrisentan, SC Treprostinil

Clinical characteristics and therapeutics at time of catheterization for the nine patients who underwent multiple catheterizations and sample collections during the study period. CDH, congenital diaphragmatic hernia. CHD, congenital heart disease. INH, inhaled. PO, per os. SC, subcutaneous. WSPH, World Symposium on Pulmonary Hypertension

## Data Availability

The data discussed in this publication have been deposited in NCBI’s Gene Expression Omnibus and are accessible through GEO Series Accession No. GSE280413 (https://www.ncbi.nlm.nih.gov/geo/query/acc.cgi?acc=GSE280413) (92). Additional clinical data are available upon request from the corresponding author.
