# Supplementary figures and images for: MicroRNA profiling to inform disease classification, severity, and treatment response in pediatric pulmonary hypertension
Source: Am J Physiol Heart Circ Physiol. Author manuscript; Available in PMC 2025 May 14. (PMC12077658; doi:10.1152/ajpheart.00622.2024)

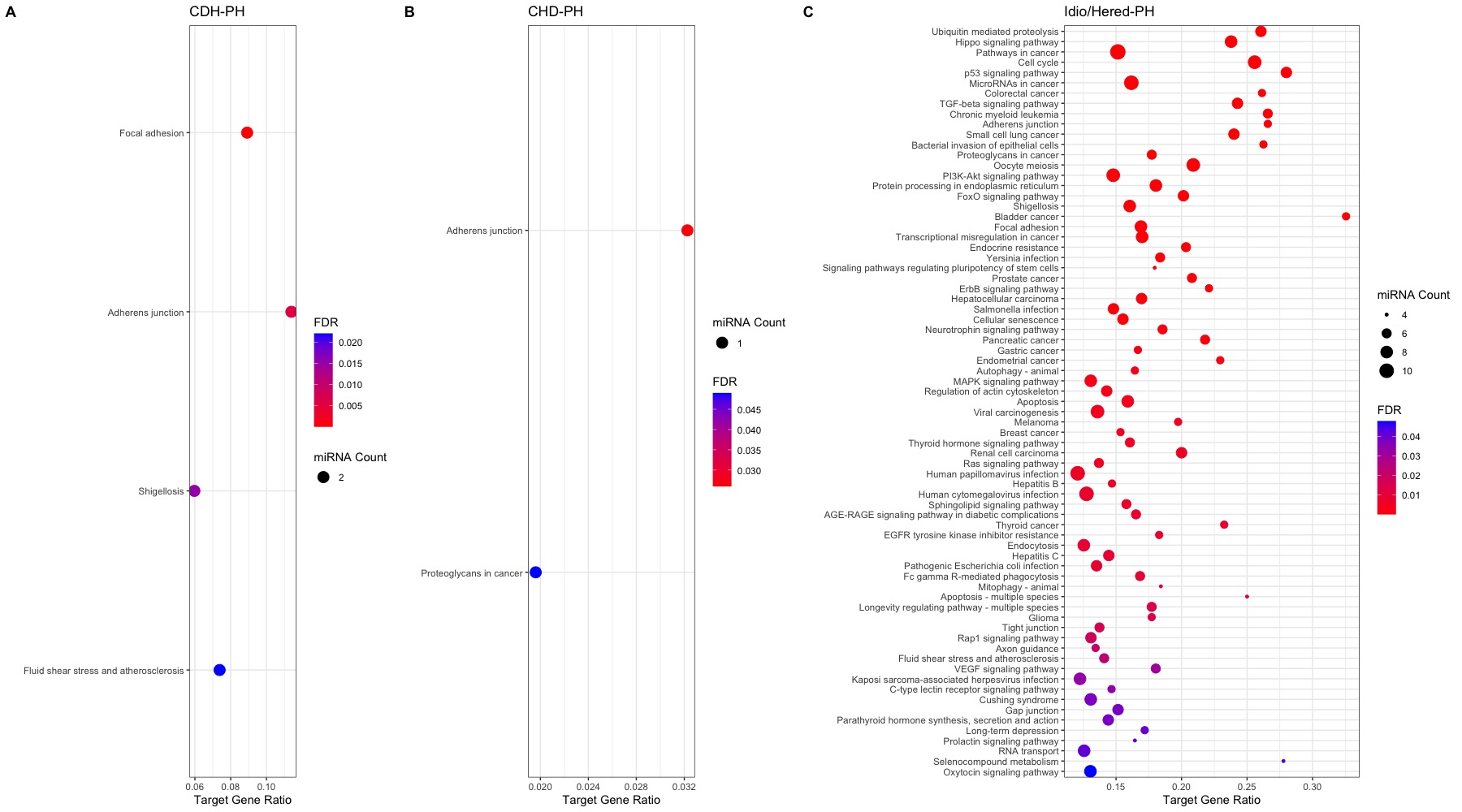

Supplement: Supplemental Figure 1 [file NIHMS2076636-supplement-Supplemental_Figure_1.tiff]
